# ‘Friendly allies in raising a child’: a survey of men and women seeking elective co-parenting arrangements via an online connection website

**DOI:** 10.1093/humrep/dev120

**Published:** 2015-06-03

**Authors:** V. Jadva, T. Freeman, E. Tranfield, S. Golombok

**Affiliations:** 1 Centre for Family Research, University of Cambridge, Free School Lane, Cambridge CB2 3RF, UK; 2 Pride Angel,www.prideangel.com

**Keywords:** elective co-parenting, online connection website, sexual orientation, gender, motivation

## Abstract

**STUDY QUESTION:**

What are the characteristics, motivations and expectations of men and women who search for a co-parent online?

**SUMMARY ANSWER:**

Male and female prospective co-parents differed in terms of their motivations, choice of co-parent and expectations of co-parenting, while differences according to sexual orientation were less marked.

**WHAT IS KNOWN ALREADY:**

Very few studies have addressed the experiences of elective co-parents, i.e. men and women who are not in a relationship with each other creating and raising a child together. No study has examined the motivations and experiences of those who seek co-parents online.

**STUDY DESIGN, SIZE AND DURATION:**

An online survey was completed by 102 participants (61 men, 41 women) who were members of Pride Angel, an online connection website that facilitates contact between people looking for someone with whom to have a child. The survey was live for 7 weeks.

**PARTICIPANTS/MATERIALS, SETTING, METHODS:**

Details of the survey were emailed to all members of Pride Angel. The survey obtained data on participants' demographic characteristics, motivations, choice of co-parent and expectations of co-parenting. Data were analysed to examine differences by gender and by sexual orientation within each gender.

**MAIN RESULTS AND THE ROLE OF CHANCE:**

Approximately one-third of men and one half of women seeking co-parenting arrangements were heterosexual. The majority (69, 68%) of participants were single, although significantly more gay and bisexual men (15, 36%) and lesbian and bisexual women (11, 55%) had a partner compared with heterosexual men (4, 20%) and heterosexual women (2, 12%), respectively. Overall, the most important motivation for seeking co-parenting arrangements was in order for both biological parents to be involved in the child's upbringing. Co-parents were looking for someone with a good medical history. Most female co-parents expected the child to live with them, whereas male co-parents either wished the child to reside with the mother or to live equally in both households. A higher proportion of gay and bisexual men than heterosexual men wanted daily contact with the child.

**LIMITATIONS, REASONS FOR CAUTION:**

Although this study presents data from the largest sample of elective co-parents to date, the main limitations were the low response rate and that only members of one website were approached. The findings may not be representative of all potential elective co-parents.

**WIDER IMPLICATIONS OF THE FINDINGS:**

This study provides important insights into the new phenomenon of elective co-parenting. With the increasing use of assisted reproductive technologies and the diversification of family forms, a growing number of people are seeking co-parenting arrangements to have children. While up until now, elective co-parenting has been principally associated with the gay and lesbian community, this study shows that, with the rise of co-parenting websites, increasing numbers of heterosexual men and women are seeking these types of parenting arrangements. This study generates the first findings on the expectations and motivations of those who seek co-parents online and examines whether these differ according to gender and sexual orientation. Future studies are needed to assess the impact of this new form of parenting on all involved, particularly the children.

**STUDY FUNDING/COMPETING INTEREST(S):**

This study was supported by the Wellcome Trust (097857/Z/11/Z). Erika Tranfield is the co-founder of the website Pride Angel, the remaining authors have no conflicts of interest to declare.

## Introduction

Elective co-parenting is a relatively new phenomenon, whereby a man and a woman who are not married, cohabiting or involved in a sexual relationship with each other have a child together and typically raise the child in separate households. This type of co-parenting differs from other uses of the term co-parenting. For example, the term ‘co-parenting’ has been used in the psychological literature to describe the extent to which parents collaborate together in raising their child. In addition, in studies of parenting by lesbian couples the non-birth mother is sometimes referred to as the co-parent or co-mother. Elective co-parenting has been more prevalent among gay men and lesbian women. However, there has recently been an increase in co-parenting arrangements among heterosexual men and women. Co-parenting can also include parents of different sexual orientation coming together to raise a child (Erera and Segal-Engelchin, 2014). Often the biological parents have partners, resulting in multiple adults planning the pregnancy and raising the child collectively (Herbrand, 2008; Smietana *et al.*, 2014).

Although elective co-parenting arrangements involving gay men and lesbian women have occurred for many years (Patterson, 1992), the way in which prospective co-parents may search for reproductive partners has changed. This is related in part to the rise of Internet websites dedicated to facilitating contact between individuals who want to meet people with the common aim of having a child. Such websites have made elective co-parenting accessible to large numbers of people, including both single people and those with partners. Furthermore, these websites are not restricted to lesbian, gay or bisexual (LGB) individuals; heterosexual men and women can also become members and contact potential reproductive partners who may be of a different sexual orientation to themselves. Given the global nature of these websites, contact can be made between individuals who reside in different countries, who need to negotiate how and where they raise their child.

It is unclear what the impact of elective co-parenting will be for the adults and children involved. In terms of living arrangements, these children are similar to those who have experienced parental divorce or separation and find themselves being raised by biological parents who live in different households, and sometimes by stepparents as well. There is a large research literature showing that children in single-parent families following parental separation or divorce (Hetherington and Stanley-Hagan, 1999; Amato, 2000, 2001, 2005; Pryor and Rogers, 2001; Coleman and Glenn, 2009) and in stepfamilies (Dunn *et al.*, 1998; Hetherington and Clingempeel, 1992; Dunn *et al.*, 2000, 2001; Hetherington and Stanley-Hagan, 2002) are more likely to show difficulties in their relationships with their parents, particularly with their non-resident parents and stepparents, and are at greater risk of developing emotional and behavioural problems, than are children in intact families. However, children from elective co-parenting arrangements will not necessarily experience the negative factors associated with parental separation or divorce, such as marital conflict, separation from a parent with whom they shared their daily lives, a drop in household income, parental distress or, for those whose custodial parent remarries or cohabits with a new partner, the need to adapt to a new stepfamily.

It is difficult to predict the likely psychological consequences for children born through elective co-parenting. There is broad consensus within the field of developmental psychology that the quality of the relationship between parents is strongly associated with children's psychological development and wellbeing, such that a close, affectionate and supportive relationship between parents provides a sense of emotional security for children and fosters positive psychological adjustment (Cummings and Davies, 2010; Reynolds *et al.*, 2014). As elective co-parents do not have a pre-existing relationship and are not a ‘couple’ in the conventional sense of the word, such qualities may be lacking from their relationship, meaning that children born to co-parents may be at an increased risk for psychological problems. On the other hand, parental collaboration in raising children is associated with positive child outcomes (Feinberg and Sakuma, 2011; Feinberg *et al.*, 2012). Thus, the more that co-parents co-operate in childrearing, the less likely their children may be to experience adverse psychological consequences. Indeed, it could be argued that these children are at an advantage compared with children born to single women through donor insemination as their biological father is present in their lives.

Little is known about the characteristics and motivations of those who choose co-parenting arrangements for family building, especially heterosexual co-parents. Studies of the characteristics of co-parents have shown that they tend to be highly educated and financially secure (Segal-Engelchin *et al.*, 2005; 2012). In addition, many co-parents already have partners who are present in the child's life from the beginning. In terms of motivations, a Belgian study of nine co-parenting arrangements involving gay and lesbian adults found that co-parents were primarily motivated by a desire to have a biologically related child and for the child to know both biological parents (Herbrand, 2008). This was deemed by the co-parents as important for the child's identity and wellbeing as well as ensuring that the child had a complete medical history. Thus, the co-parents wanted a ‘conventional family’, where the child is raised by their biological mother and father (Herbrand, 2008; Smietana *et al.*, 2014). An Israeli study of 10 heterosexual women co-parenting with gay men similarly found that they wanted their child to have both a mother and a father. These women reported that in addition to fulfilling their wish to raise a family, co-parenting provided financial security and enabled the parenting burden to be shared (Segal-Engelchin *et al.*, 2012). Unlike single heterosexual women who conceive through donor insemination and parent without the involvement of a father (sometimes referred to as single mothers by choice), it was important for women choosing co-parenting that their child had a father figure (Segal-Engelchin *et al.*, 2012). Similar ‘traditional’ beliefs were also found among Israeli gay men co-parenting with heterosexual women, in that they wanted to raise a biologically related child and they believed it was in the child's best interests to have an actively involved mother and father. Alongside these traditional views, these men also held ‘new’ and ‘progressive’ views of parenting whereby they wanted to be highly involved and active in raising their child (Erera and Segal-Engelchin, 2014). Men can ascribe a variety of meanings to the relationship with their biological children which can differ between individuals (Dempsey, 2012).

Herbrand's (2008) study of Belgian gay and lesbian co-parents provides some insight into how co-parenting arrangements may be managed. The biological parents held legal parentage of the child and were the main child-care providers. The father's partner was less involved than the mother's partner, partly due to the fact that the father's partner often did not want children, and also because the children lived mainly with their mother, which resulted in fewer opportunities for child caring duties by fathers (Herbrand, 2008; Smietana *et al.*, 2014). Thus, the degree of involvement by each of the co-parents can differ and needs to be negotiated and managed by the adults concerned.

Given the increasing prevalence of co-parenting arrangements and the rise of websites, which facilitate them, it is important to better understand this growing phenomenon. The present study explored who are seeking to become co-parents and why, by examining the characteristics, motivations and expectations of people searching for co-parenting arrangements using ‘Pride Angel’ (www.prideangel.com), an online connection website that enables people to meet others for the purpose of creating a child. As elective co-parenting has been most prevalent among gay and lesbian parents and heterosexual men and women have only recently begun to have children in this way, similarities and differences were examined by sexual orientation and gender. Members of Pride Angel include sperm and egg donors, those looking for sperm and egg donors (sperm and egg recipients) and those looking for co-parents. The present paper examines data from members who were searching for co-parents.

## Materials and Methods

All Pride Angel members were sent an email about the study from the founder of Pride Angel that contained a web link directing them to the front page of the survey. This front page provided further information about the study, consent procedures, and a link to start the survey. Details of the study were also advertised on the home page of Pride Angel. The survey was live for 7 weeks from mid-February to the end of March 2014. Email invitations were sent at the beginning of the survey followed by two reminder e-mails. Participants received 10 free message credits (approximate value £10) for completing the survey. Ethical approval for this study was obtained from the University of Cambridge Psychology Research Ethics Committee.

Response rates are intrinsically difficult to calculate for online surveys. Although this survey was restricted to Pride Angel members, not all members were currently using the website and most of the emails that were sent were not opened. Online membership (i.e. those with web profiles) at the beginning of the survey was 27 650 members, comprising 17 367 registered as sperm recipients, 5299 registered as sperm donors, 866 registered as egg recipients, 547 registered as egg donors, and 3571 registered as co-parents. A total of 32 634 emails were successfully sent to all members (those with and without on-line profiles), of which 5425 emails were opened, representing 19.6% of online members and 16.6% of total members. Of those who opened the email (i.e. accessed the survey information page), 1402 (25.8%) started the survey and 1022 (18.8%) completed the survey. Of these, a total of 102 were completed by prospective co-parents, comprising 14.6% of the estimated number (i.e. 701) of prospective co-parents who opened the email. Although this proportion appears small, the sample size is larger than any other study of co-parenting and reflects the low response rates typically achieved in on-line studies compared with other survey methods such as postal questionnaires (Cook *et al.*, 2000; Nulty, 2008). An advantage of web-based studies is that they can access unique and difficult to reach populations (Wright, 2005; Hewson, 2014). In order to assess whether our sample reflected the demographic of Pride Angel members, the gender, sexual orientation and relationship status of the participants in the present study were compared with that of members of Pride Angel at the start of the survey. Similar proportions of co-parents had taken part in our survey based on sexual orientation and relationship status. However, a higher proportion of men completed our survey (60%) compared with those on the website (35%).

### Measures

The survey comprised a number of multiple choice and open-ended questions. Data were obtained on: (i) participant characteristics, including gender, sexual orientation, relationship status, number of children, ethnicity, religion, educational level, working status and country of residence; (ii) motivations for co-parenting, where participants were presented with a list of different motivations and asked to rank each one on a 5-point scale ranging from not very important to very important. Their reasons for choosing co-parenting over any other means of having a child were also obtained; (iii) choosing a co-parent, including whom they would like to co-parent with, whether they had any restrictions on whom they would like to co-parent with, and if so, what these restrictions were, which methods of conception they would consider, the length of time they would like to be in contact before trying to conceive and whether they had undergone any preparation for becoming co-parents (e.g. medical screening, counselling); (iv) expectations of co-parenting, including how they saw their relationship with the co-parent, how frequently they wanted the co-parent to see the child, where they would like the child to live, and an open-ended question about how they envisaged the relationship with the co-parent.

### Data analysis

Gender differences and differences according to sexual orientation within each gender were examined using Mann–Whitney *U*-tests for co-parents motivations and *χ*^2^ and Fisher’s exact tests for all other variables. The content of responses to open-ended questions was systematically coded into categories using the qualitative data analysis software Atlas-ti, and the most common responses were reported as frequencies.

## Results

### Participant characteristics

A total of 102 people seeking to become co-parents completed the on-line survey comprising 61 (60%) men and 41 (40%) women. Overall, one-third (38, 37%) of participants were heterosexual, 46 (45%) were lesbian/gay, 15 (15%) were bisexual and 3 (3%) selected ‘other’ or did not specify their sexual orientation. Of the heterosexual respondents, 18 (47%) were women and 20 (53%) were men. Thirty-three (32%) participants had a married/civil/cohabiting partner and 69 (68%) participants were single. A significant association was found between sexual orientation and relationship status for women (*χ*^2^ (1, *n* = 38) = 8.11, *P* < 0.005), with a higher proportion of heterosexual participants (16, 89%) compared with lesbian/bisexual participants (9, 45%) stating their relationship status to be single. The majority of participants did not have any children, with only 8 (13%) men and 7 women (18%) reporting that they did. Heterosexual men were more likely to have at least one child (6, 30%) compared with gay/bisexual men (2, 5%), Fisher’s exact = 0.012, see Table I.

**Table I:** Current relationship status and parental status of prospective co-parents by gender and sexual orientation.

	Male				Female				Total	
	Heterosexual		Gay/bisexual		Heterosexual		Lesbian/bisexual			
	*n*	%	*n*	%	*n*	%	*n*	%	*n*	%
Relationship status										
Cohabiting	1	5	12	29	1	6	7	35	21	21
Married/civil partnership	3	15	3	7	1	6	4	20	11	11
Single	13	65	26	64	15	82	9	45	63	64
Divorced/separated	3	15	0	0	1	6	0	0	4	4
Parental status										
No children	14	70	39	95	15	83	16	80	84	85
Has at least one child	6	30	2	5	3	17	4	20	15	15
Total	20	100	41	100	18	100	20	100	99	100

Participants were aged from 18 to 54 years (mean = 36, SD = 8.2). The men were significantly older (mean = 38.1, SD = 8.5) than the women (mean = 33.2, SD = 6.9) (*t* (100) = 3.05, *P* = 0.003). Examination of the age of participants by sexual orientation within each gender showed that the heterosexual men appeared to be slightly older (mean = 41.1, SD = 8.9) than the gay and bisexual men (mean = 36.6, SD = 6.7) and the heterosexual women appeared older (mean = 35.3, SD = 6.7) than the lesbian and bisexual women (mean = 31.9, SD = 7.03). However, these differences were not statistically significant.

Most participants (68, 67%) classified their ethnicity as White, with the remaining participants being Black (14, 14%), Asian (7, 7%), Mixed Race (8, 8%) or ‘other’ (3, 3%). In terms of religion, the majority of participants stated either ‘no religion’ (44, 43%) or Christian (40, 39%); other participants were Jewish, Hindu, Buddhist, Muslim (<4% in each case) or ‘other’ (7, 7%). In terms of education, most had a University degree or higher (68, 67%). The majority of participants were working full-time (72, 71%), with the remainder being equally split between those who were working part-time (15, 15%) and those who were not working (15, 15%). The largest proportion of participants lived in the UK (56, 55%), with 16 (16%) living in the USA and 7 (7%) living in Australia. Other countries of residence were Canada, Cyprus, Germany, Indonesia, Japan, Lebanon, Mexico, Nigeria, Portugal, Serbia, Singapore, South Africa, Spain, Sweden and Switzerland (<2% in each case).

Thirty-two (31%) participants had made contact with potential co-parents through Pride Angel. Only one woman had had a child through a co-parenting arrangement.

### Motivations for co-parenting

Overall, the motivations for seeking a co-parenting arrangement that were ranked as most important were ‘Wanting the child to know both biological parents’ and ‘Wanting to know the person who provides the sperm/egg to create the child’. The median values for the importance of each motivation by gender and sexual orientation are given in Table II. Comparisons by gender found women to rate ‘I am getting older’ (*U* = 682.0, *P* = 0.021) and ‘I am single’ (*U* = 416.5, *P* = 0.002) as more important than men. Men rated ‘To pass on my genes’ (*U* = 585, *P* = 0.031) as more important than women. No other differences by gender were found. Comparisons by sexual orientation within each gender found that lesbian and bisexual women rated ‘Co-parenting is an ideal situation for bringing up a child’ (*U* = 125.0, *P* = 0.022) and ‘Family/friends have used sperm/egg donation’ (*U* = 19.5, *P* = 0.035) as more important than heterosexual women. No other differences by sexual orientation were found.

**Table II:** Motivations to co-parent by gender and sexual orientation.

	Male				Female				Total	
	Heterosexual		Gay/bisexual		Heterosexual		Lesbian/bisexual			
	Median (interquartile range)	*n*	Median (interquartile range)	*n*	Median (interquartile range)	*n*	Median (interquartile range)	*n*	Median (interquartile range)	*n*
I would like my child to know both their ‘biological’ parents	4.5 (1.75)	20	5 (1)	41	5 (1)	18	5 (1)	19	5 (1)	98
I want to know the person who provides the sperm/egg to create my child	4 (2)	16	5 (1)	40	5 (1)	17	5 (1)	18	5 (1)	91
Co-parenting is an ideal situation for bringing up a child	4 (2)	19	4 (1)	40	3 (1)	18	4 (1.25)	18	4 (2)	95
I want the person who provides the sperm/egg to be involved in my child's upbringing	4 (2)	17	5 (2)	41	5 (1)	17	4 (2)	19	4 (2)	94
I do not want to parent alone	4 (1.5)	17	4 (3)	39	4 (1.5)	17	3 (1.5)	18	4 (2)	91
I am getting older	4 (2)	16	4 (2)	35	5 (1)	18	4 (2)	19	4 (2)	88
I am single	4 (1.75)	16	3 (1)	32	5 (1)	16	4 (2)	13	4 (2)	77
To pass on my genes	3 (1.5)	17	4 (2)	37	3.5 (1.25)	14	3 (2.75)	16	4 (1.75)	84
To share the financial cost of parenting	3 (1)	16	3 (1)	38	4 (1)	17	4 (3.75)	16	3 (1)	87
No other option available	3 (1)	16	3 (1.75)	32	4 (2)	17	3 (2)	11	3 (1)	76
No reason not to	3 (0.25)	10	3 (2)	33	3.5 (1.75)	14	3 (1)	14	3 (1)	71
My partner does not want a child	3 (0.25)	10	3 (1)	19	4 (0.75)	4	3 (3.25)	6	3 (1)	39
Family/friends have used sperm/egg donation	3 (0)	11	2 (2.75)	20	1 (2.5)	8	3 (1)	11	3 (3)	50
I do not wish to have a child within a relationship	3 (2)	16	3 (2)	27	2 (3)	10	2 (2)	16	2 (2)	69

Scale ranged from 1‘not at all important’ to 5 ‘very important’.

As not all participants ranked each of the motivations, the sample size comprises those who answered the question.

Sixty-five (64%) participants responded to open-ended questions asking why they had chosen co-parenting over other means of having a child, including sperm/egg donation and adoption. The most frequent response (*n* = 20) related to children having both parents in their lives. For example,

Co-parenting allows the child to know and love both parents. (Bisexual female participant)

[Co-parenting is] closest to natural mum and dad family upbringing. (Gay male participant)

### Choosing a co-parent

As given in Table III, most participants were looking for a single co-parent or a gay/lesbian couple. A significant gender difference was found for the option ‘couple – any sexual orientation’, indicating that men were more likely than women to select this option (Fisher's exact = 0.02). Comparisons by sexual orientation within each gender revealed that heterosexual men were more likely than gay and bisexual men to be looking for a single heterosexual woman (*χ*^2^ (1, *n* = 61) = 6.959, *P* < 0.001) and gay and bisexual men were more likely than heterosexual men to be looking for a single woman of any sexuality (*χ*^2^ (1, *n* = 61) = 7.25 *P* < 0.001). In addition, heterosexual women were more likely than lesbian and bisexual women to be looking for a single heterosexual man (*χ*^2^ (1, *n* = 38) = 12.685, *P* < 0.001). Overall regardless of gender and sexual orientation, single co-parents were less likely to be looking for a couple to co-parent with than co-parents with a partner (*χ*^2^ (1, *n* = 102) = 4.144 *P* < 0.05), and a non-significant trend was found showing that single co-parents were more likely to be looking for a single co-parent than those with a partner (*χ*^2^ (1, *n* = 102) = 3.494 *P* = .062).

**Table III:** Choosing a co-parent by gender and sexual orientation.

	Male				Female				Total	
	Heterosexual		Gay/bisexual		Heterosexual		Lesbian/bisexual			
	*n*	%	*n*	%	*n*	%	*n*	%	*n*	%
Who are you looking to co-parent with?^a^										
No preference	2	10	13	32	2	11	2	10	19	19
Couple—gay/lesbian	8	40	18	44	7	39	12	60	45	45
Couple-heterosexual	4	20	5	12	2	11	2	10	13	13
Couple—any sexual orientation	1	5	7	17	0	0	0	0	8	8
Single—gay/lesbian	11	55	15	37	7	39	11	55	44	44
Single—heterosexual	14	70	14	34	14	78	4	20	46	46
Single—any sexual orientation	8	40	19	46	7	39	8	40	42	42
Other	0	0	1	2	0	0	2	10	3	3
Do you have any restrictions about who you would enter into a co-parenting arrangement with?										
Yes	11	55	27	66	16	88	11	55	65	66
No	6	30	7	17	1	6	3	15	17	17
Not sure	3	15	7	17	1	6	6	30	17	17
What aspect of the co-parent are these restrictions based on?^a^										
Medical history	9	45	18	44	12	67	9	45	48	48
Expectations of co-parenting	4	20	19	46	12	67	8	40	43	43
Whether co-parent would be a good parent	8	40	17	42	10	56	8	40	43	43
Ideas about parenting	6	30	16	39	10	56	9	45	41	41
Reasons for wanting a child	6	30	18	44	9	50	8	40	41	41
Personality	6	30	16	39	7	39	8	40	37	37
Physical appearance	8	40	16	39	6	33	4	20	34	34
Reasons for wanting to co-parent	5	25	14	34	9	50	6	30	34	34
Religion	4	20	14	34	9	50	6	30	33	33
Age	6	30	14	27	7	39	6	30	33	33
Number of children co-parent already has	4	20	12	29	9	50	4	20	29	29
Current place of residence	6	30	13	32	5	28	4	20	28	28
Financial situation	7	35	11	27	6	33	4	20	28	28
Race/ethnicity	2	10	9	22	11	61	5	25	27	27
Education	6	30	11	27	5	28	5	25	27	27
Marital status	3	15	13	32	3	15	3	17	22	22
Previous fertility problems	5	25	7	17	2	11	5	25	19	19
Sexual orientation	7	35	7	17	2	11	2	18	18	18
Family history	5	25	8	20	2	11	3	15	18	18
Number of children co-parent would like to have	3	15	6	15	5	28	3	15	17	17
Occupation	3	15	6	15	2	11	3	15	14	14
Personal interests	4	20	7	17	1	6	1	5	13	13
Nationality	1	5	3	7	5	28	0	0	9	9
Other, please specify	0	0	1	2	1	6	0	0	2	2
Which ways of conceiving would you consider?^a^										
At a clinic	15	75	35	85	14	78	14	70	78	78
Home insemination/artificial insemination	14	70	37	90	15	83	18	90	84	84
Sexual intercourse/natural insemination	14	70	9	22	8	44	3	15	34	34
Other	1	5	1	2	0	0	1	5	3	3
How long would you like to be in contact with the co-parent(s) before you start trying to conceive a child together?										
I do not mind	10	50	12	29	3	17	4	20	29	29
≤1 week	0	0	0	0	0	0	1	5	1	1
2–4 weeks	2	10	2	10	2	11	4	20	10	10
1–3 months	6	30	6	30	4	22	3	15	19	19
3–6 months	0	0	10	24	6	33	0	0	16	16
6 months to 1 year	2	10	9	22	2	11	5	25	18	18
1–2 years	0	0	2	5	0	0	2	10	4	4
≥2 years	0	0	1	2	1	6	1	5	3	3

^a^Participants could select more than one response.

Sixty-five (66%) participants had restrictions on whom they would enter into a co-parenting arrangement with. These restrictions were most commonly based on ‘medical history’ and ‘expectations of co-parenting’ followed by ‘whether the co-parent would be a good parent’ (see Table III for a full list of the categories selected). A gender difference was found for ‘race/ethnicity’ (*χ*^2^ (1, *n* = 68) = 5.084, *P* < 0.05), showing that a significantly higher proportion of women than men selected this as a restriction although the number of people selecting this option was small (*n* = 27).

Table III shows the methods of conception that co-parents would consider to conceive their child. In terms of their most preferred method, the highest proportion of participants (26, 42.3% men and 21, 51.2% women) stated ‘home insemination (i.e. by “artificial insemination”)’, followed by ‘at a clinic’ (20, 32.7% men and 13, 31.7% women). ‘Sexual intercourse (i.e. by “natural insemination”)’ was the least preferred method (13, 21.3% men and 7, 17.1% women). One man selected ‘other’ explaining that the method would depend on the co-parent. No differences were found between men and women in the methods they would use. Comparisons by sexual orientation within each gender found that a higher proportion of heterosexual men than gay and bisexual men selected the option ‘natural insemination’ (*χ*^2^ (1, *n* = 61) = 13.213, *P* < 0.001); similarly a higher proportion of heterosexual women than lesbian and bisexual women selected this option (*χ*^2^ (1, *n* = 38) = 3.993, *P* < 0.05).

As Table III shows, the length of time participants wished to be in touch before they started trying to conceive a child varied greatly, ranging from one participant stating less than a week to three participants stating more than 2 years. Almost one-third of participants (29, 29%) specified that they did not mind, though the majority (53, 53%) favoured between 1 and 12 months. Comparisons by gender found no differences between men and women. However, comparisons by sexual orientation within each gender found a significant difference between the responses of heterosexual and gay or bisexual men (Fisher's exact = 0.015), reflecting a larger proportion of heterosexual men endorsing the option ‘don't mind’. No differences were found between heterosexual women and lesbian or bisexual women.

Fifty-five (53.9%) participants (35 men and 20 women) had carried out medical screening in preparation for becoming a co-parent. Of these, 14 (25%) (7, 20% men and 7, 35% women) had received counselling, 13 (24%) (7, 20% men and 7, 35% women) had sought medical advice and 10 (18%) (4, 11% men and 6, 30% women) had drawn up a legal agreement. Comparisons by sexual orientation within each gender revealed that a higher proportion of lesbian and bisexual women than heterosexual women had drawn up a legal agreement (Fisher's exact = 0.048).

### Expectations of co-parenting

Fifty-three participants (30 men and 23 women) provided responses to the open-ended question, ‘Please describe how you see your relationship with the co-parent’. Within those responses, the most common terms used related to friendships, e.g. ‘friendship’, or ‘friendly’ (11, 37% men and 11, 48% women). Other common terms used to describe the relationship were as a ‘partnership’ or as ‘equals’ (8, 27% men and 2, 9% women). A smaller proportion stated that the relationship had to be ‘good/positive’ or ‘civil’ (6, 20% men and 3, 13% women).

Table IV shows how often participants wished the co-parent to see the child once the child was born. There was a significant effect for gender (Fisher's exact = 0.000), with the large majority of women selecting ‘everyday’ compared with just under half of men choosing this option. Comparisons by sexual orientation within each gender found a higher proportion of gay and bisexual men than heterosexual men wishing to see the child every day (Fisher's exact = 0.003). A significant gender difference was also found for where the participant wanted the child to live (Fisher's exact = 0.000). More men than women wanted the child to live in both homes equally whereas more women than men wanted the child to live with them all the time. A significant difference according to sexual orientation was found for where the participant wanted the child to live for men only (Fisher’s exact = 0.042). Gay and bisexual men were more certain of where they wanted the child to live (either with them or with the co-parents), whereas heterosexual men were more likely to select ‘don't know’ as an option.

**Table IV:** Expectations of co-parenting by gender and sexual orientation.

	Male				Female				Total	
	Heterosexual		Gay/Bisexual		Heterosexual		Lesbian/bisexual			
	*n*	%	*n*	%	*n*	%	*n*	%	*n*	%
How often would you like the co-parent(s) to see the child once they are born?										
Every day	5	25	18	44	5	28	4	20	32	32
Once a week	3	15	11	27	5	28	3	15	22	22
Once a fortnight	2	10	3	7	1	6	3	15	9	9
Once a month	1	5	1	2	1	6	4	20	7	7
Once a year	1	5	0	0	1	6	0	0	2	2
Less than once a year	0	0	0	0	2	11	1	5	3	3
Not sure	8	40	8	20	3	17	5	25	24	24
How often would you like to see the child?										
Every day	5	26	24	59	17	94	18	95	64	64
Once a week	5	26	10	24	0	0	0	0	15	15
Once a fortnight	0	0	3	7	0	0	0	0	3	3
Once a month	1	5	2	5	0	0	0	0	3	3
Once a year	0	0	0	0	0	0	0	0	0	0
Less than once a year	0	0	0	0	0	0	0	0	0	0
Not sure	8	42	2	5	1	6	1	5	12	12
Where would you like the child to live?										
My home all of the time	0	0	1	3	9	50	10	50	20	20
My home most of the time	1	5	5	13	4	22	6	30	16	16
My and co-parent's home equally	9	45	18	45	4	22	2	10	59	59
Co-parent's home most of the time	2	10	12	30	0	0	0	0	14	14
Co-parent's home all of the time	3	15	3	8	0	0	0	0	6	6
Do not know	5	25	1	3	1	6	2	10	9	9

## Discussion

This is the first study to examine the characteristics, motivations and expectations of men and women using an online connection website to find people to have children with as ‘co-parents’. Both men and women were seeking such co-parenting arrangements. While the majority of prospective co-parents were currently single, almost one-third had a partner, most of whom were LGB. That the sample varied in terms of sexual orientation reinforces findings from other studies that co-parenting is not restricted to the lesbian and gay community. Indeed, findings from the current study suggest that potential co-parents are not a homogenous group. Co-parents were also found to vary in age, with the youngest aged 18 years to the eldest being in their mid-50s with the average age of most participants being early- to mid-30s. Prospective co-parents were also highly educated, a similar finding to that reported elsewhere (Segal-Engelchin *et al.*, 2012; Erera and Segal-Engelchin, 2014). The majority of the sample classified their ethnicity as white, with over half living in the UK, although people of different nationalities and from different ethnic backgrounds were also seeking co-parenting arrangements.

This study found that those looking to become co-parents are mainly motivated by a desire to have both biological parents involved in the upbringing of the child, a finding that has also been observed in previous studies (Herbrand, 2008; Segal-Engelchin *et al.*, 2012; Erera and Segal-Engelchin, 2014; Smietana *et al.*, 2014). This desire reflects the traditional significance placed on genetic relatedness as defining parenthood as well as on the two opposite-sex parent (i.e. mother and father) family model irrespective of the sexual orientation of the parents. The importance attributed to genetic relatedness for those planning to have children using assisted reproduction within ‘non-traditional’ parenting arrangements has been found to vary according to individual circumstance (Freeman, 2014). For example, when comparing gay men seeking to have children via co-parenting, surrogacy and adoption arrangements, the value of genetic connections in defining parent-child relationships was most clearly expressed in the co-parenting group (Smietana *et al.*, 2014).

Individuals looking for co-parents were seeking a single person or a lesbian or gay couple. They were also often searching for someone with a good medical history. Women were more likely than men to be looking for someone of a particular race or ethnicity. This may have been a result of them wishing to match the co-parent to their partner's race or ethnic background, a finding similar to that found in studies of lesbian and heterosexual couples choosing a sperm donor (Scheib *et al.*, 2000; Nordqvist, 2010). However, analysis of the relationship status of women who selected this option revealed that most were single and not in a relationship, suggesting that they may wish to match the co-parent to their own race or ethnic background. It is important to note that the number of cases that selected this response was small and this restriction was not important to most elective co-parents in the study. It is also unclear why this would be of more importance to women than to men. Differences were also found between men and women in regard to where the child would live and how frequently they planned to see the child, with most women wanting the child to live with them and half of men wanting the child to live equally with them and the co-parent(s). Furthermore, differences were identified according to sexual orientation, with a higher proportion of gay and bisexual men than heterosexual men wanting daily contact with the child. In the open-ended responses, prospective co-parents explained that contact and living arrangements will be negotiated with each other, and that these may change as the child grows older. These living arrangements reflect wider social patterns of gender differences in child care among heterosexual couples who live together where women carry out more child-care duties than do men (Craig and Mullan, 2011) and suggest that co-parenting arrangements tend to replicate the traditional family, not only in structure but also in terms of gender roles.

A particular concern arising from these findings is the length of time prospective co-parents planned to be in contact with each other prior to attempting conception. Most expected to be in contact for a few months which raises the question of whether this allows sufficient time to establish a sustainable co-parenting relationship. Longitudinal studies of co-parenting arrangements would enable a better evaluation of whether meeting via the Internet and knowing each other for a few months can lead to the type of co-parenting relationships that the participants envisioned. The open-ended responses revealed that participants' expectations of co-parenting were idealised in that they wanted a friendship with the co-parent and a happy loving family in which all parents were accepted and the child was loved. Where more than two parents are involved in the upbringing of the child, each parent's role and responsibility would need to be acknowledged by the child, the other parents and their wider social network. Ultimately only two parents would be able to hold legal responsibility for the child, although co-parenting arrangements have prompted discussion of whether more than two parents should hold legal parentage (Cutas, 2011).

A particular strength of the current study was that most participants were in the process of seeking co-parents and thus their responses were not reliant on recall bias which has been a limitation of previous studies (Segal-Engelchin *et al.*, 2012; Erera and Segal-Engelchin, 2014). In addition, the survey was carried out anonymously and thus respondents may have felt more able to provide open and honest answers. However, survey methodologies have limitations, including the inability to probe and question the responses given. Another limitation was the low response rate, although it has to be remembered that only one-fifth of online members opened the email invitation. Participation rates for on-line surveys are typically low and hard to calculate (Hewson, 2014), and it is unclear whether those who took part in the current study were representative of all members of co-parents on the website. However, similar proportions were found between participants in the study and Pride Angel members as a whole in terms of sexual orientation and relationship status. The higher proportion of male participants' in this study increases the generalizability of the findings regarding the motivations and experiences of prospective male co-parents. Another limitation is that it is not known whether members of Pride Angel are comparable with members of other similar sites. However, Pride Angel is one of the largest and most established sites of its kind in the UK. Furthermore, the findings replicate those from studies of co-parents in Belgium and Israel suggesting some commonalities between co-parents from different countries.

While this survey provides new insights and confirms previous findings on elective co-parenting, it is important to conduct prospective studies of co-parenting arrangements in order to better understand how this takes place in practice, to what extent co-parents' expectations are met and, most crucially, the consequences of this new form of parenting for the psychological development and wellbeing of children raised in this way.

## Authors’ roles

All authors were involved in the design of this study. Erika Tranfield assisted with the recruitment of participants. All other authors were involved in the analysis and interpretation of data. This manuscript was drafted by V.J. and has been approved by all authors.

## Funding

This study was supported by the Wellcome Trust (097857/Z/11/Z). Funding to pay the Open Access publication charges for this article was provided by the University of Cambridge.

## Conflict of interest

E.T. is Director and Co-founder of the website www.prideangel.com. The other authors have no conflicts of interest to declare.
